# Pathologic Substrates of Structural Brain Network Resilience and Topology in Parkinson Disease Decedents

**DOI:** 10.1212/WNL.0000000000209678

**Published:** 2024-07-23

**Authors:** Irene Frigerio, Tommy A.A. Broeders, Chen-Pei Lin, Maud M.A. Bouwman, Ismail Koubiyr, Frederik Barkhof, Henk W. Berendse, Wilma D.J. Van De Berg, Linda Douw, Laura E. Jonkman

**Affiliations:** From the Department of Anatomy and Neurosciences (I.F., T.A.A.B., C.-P.L., M.M.A.B., I.K., W.D.J.V.D.B., L.D., L.E.J.), and Department of Radiology and Nuclear Medicine (F.B.), Amsterdam UMC location Vrije Universiteit Amsterdam, the Netherlands; Institutes of Neurology and Healthcare Engineering (F.B.), University College London, United Kingdom; and Department of Neurology (H.W.B.), Amsterdam UMC location Vrije Universiteit Amsterdam, the Netherlands.

## Abstract

**Background and Objectives:**

In Parkinson disease (PD), α-synuclein spreading through connected brain regions leads to neuronal loss and brain network disruptions. With diffusion-weighted imaging (DWI), it is possible to capture conventional measures of brain network organization and more advanced measures of brain network resilience. We aimed to investigate which neuropathologic processes contribute to regional network topologic changes and brain network resilience in PD.

**Methods:**

Using a combined postmortem MRI and histopathology approach, PD and control brain donors with available postmortem in situ 3D T1-weighted MRI, DWI, and brain tissue were selected from the Netherlands Brain Bank and Normal Aging Brain Collection Amsterdam. Probabilistic tractography was performed, and conventional network topologic measures of regional eigenvector centrality and clustering coefficient, and brain network resilience (change in global efficiency upon regional node failure) were calculated. PSer129 α-synuclein, phosphorylated-tau, β-amyloid, neurofilament light-chain immunoreactivity, and synaptophysin density were quantified in 8 cortical regions. Group differences and correlations were assessed with rank-based nonparametric tests, with age, sex, and postmortem delay as covariates.

**Results:**

Nineteen clinically defined and pathology-confirmed PD (7 F/12 M, 81 ± 7 years) and 15 control (8 F/7 M, 73 ± 9 years) donors were included. With regional conventional measures, we found lower eigenvector centrality only in the parahippocampal gyrus in PD (*d* = −1.08, 95% CI 0.003–0.010, *p* = 0.021), which did not associate with underlying pathology. No differences were found in regional clustering coefficient. With the more advanced measure of brain network resilience, we found that the PD brain network was less resilient to node failure of the dorsal anterior insula compared with the control brain network (*d* = −1.00, 95% CI 0.0012–0.0015, *p* = 0.018). This change was not directly driven by neuropathologic processes within the dorsal anterior insula or in connected regions but was associated with higher Braak α-synuclein staging (*r*_s_ = −0.40, *p* = 0.036).

**Discussion:**

Although our cohort might suffer from selection bias, our results highlight that regional network disturbances are more complex to interpret than previously believed. Regional neuropathologic processes did not drive regional topologic changes, but a global increase in α-synuclein pathology had a widespread effect on brain network reorganization in PD.

## Introduction

Parkinson disease (PD) is a neurodegenerative disorder characterized by α-synuclein accumulation in specific neurons.^1^ The characteristic spatial pattern of α-synuclein aggregation^1^ suggests that α-synuclein spreads across trans-synaptically connected brain regions,^2^ leading to neuronal loss and disruption of connecting white matter (WM) pathways.^3^ Advances in diffusion-weighted imaging (DWI) and graph theory have allowed the study of these WM pathways as interconnected structural brain networks,^4^ showing altered regional network topologic properties in PD.^5-7^ Graph theory in combination with computational manipulations of the structural brain network can also quantify the ability of the brain network to withstand external perturbations,^4,8,9^ giving a measure of brain resilience. Brain resilience is essential in neurodegenerative diseases because the brain needs to cope with neuropathologic accumulation while retaining its functional properties and performance.^10^ While brain resilience to tau neuropathologic burden was shown to be altered and linked to age and sex in Alzheimer disease (AD),^11^ studies on structural brain network resilience in PD have not been performed yet.

Besides α-synuclein accumulation, AD copathology, including β-amyloid (Aβ) plaques^12^ and phosphorylated-tau (p-tau) neurofibrillary tangles (NFT),^13^ is often present in PD.^14^ Moreover, neuroaxonal^15^ and synaptic degeneration^16^ also occur across several cortical regions in PD. Studies combining MRI and CSF show that α-synuclein and tau levels are associated with changes in global network topologic properties in PD.^17^ However, which (combination) of these neuropathologic processes contributes to regional changes in network topologic properties and brain network resilience is unclear. Understanding the neuropathologic drivers of network topology and resilience could give us further insights into the neuropathologic underpinnings of WM disconnections in PD.

Using a unique combination of within-subject postmortem in situ MRI and histopathology,^18^ this study aimed to investigate which neuropathologic measures, including neuropathology burden and neuroaxonal and synaptic degeneration, contribute to regional network topologic and brain network resilience differences in PD and control decedents.

## Methods

### Donor Inclusion

In collaboration with the Netherlands Brain Bank (NBB)^19^ and Normal Aging Brain Collection Amsterdam (NABCA),^18^ we included a total of 34 brain donors, of which 19 were PD brain donors, based on clinical presentation.^20^ Age at symptom onset and disease duration (age at death minus age at diagnosis) were extracted from the clinical files of the donors. Clinical Dementia Rating (CDR) scores^21^ were available for 11 of the 19 PD donors. The PD cases presented with a spectrum of cognitive impairment (CDR ranged from 0.5 to 3), among which 7 cases presented with dementia.^22^ An expert neuropathologist confirmed the clinical diagnosis, according to the international guidelines of the Brain Net Europe II consortium.^19^ In addition, 15 non-neurologic control donors with available DWI and DWI reference scans were included from NABCA.^18^ For donor characteristics, see eTable 1.

### Standard Protocol Approvals, Registrations, and Patient Consents

All donors signed an informed consent form for brain donation and the use of material and clinical information for research purposes. The Medical Ethical Committee of Amsterdam UMC, Vrije Universiteit Amsterdam, has approved the procedures for brain tissue collection of NBB and NABCA.

### Postmortem In Situ MRI Acquisition

**Figure 1:**
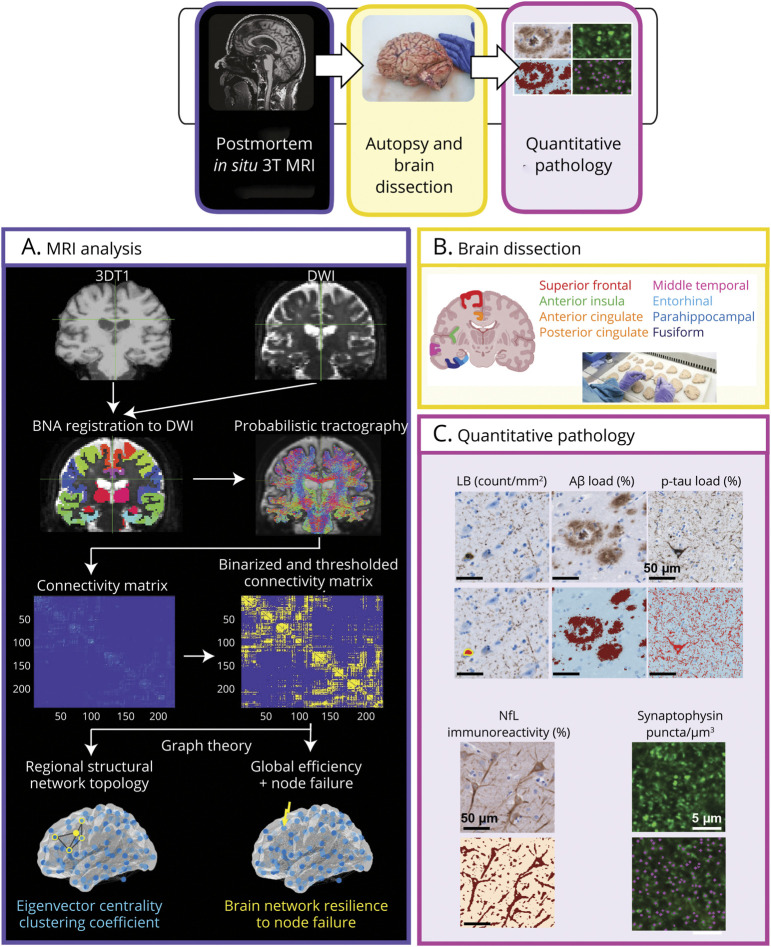
Workflow Postmortem in situ MRI (3D-T1 and DWI) of the brain was performed in consenting brain donors.^18^ (A) Gray matter was parcellated on 3D-T1 scans based on the Brainnetome Atlas, and probabilistic tractography was performed on DWI. A connectivity matrix was derived, which was binarized and thresholded. Finally, regional eigenvector centrality and clustering coefficient and brain network resilience to node failure were calculated with the Brain Connectivity Toolbox and Matlab. (B) After the MRI, autopsy was performed, and 8 cortical regions were dissected from the right hemisphere. (C) Brain tissue was processed for immunohistochemistry against pSer129 α-synuclein, p-tau, and Aβ, and neuropathology load was quantified using QuPath. Neuroaxonal degeneration was assessed by NfL immunoreactivity, quantified using QuPath. Synaptic density was assessed by synaptophysin+ puncta per μm^3^, quantified with NIS elements. The correlations between MRI and pathology outcome measures were investigated with rank-based estimation tests with age, sex, and postmortem delay as covariates. Scale bar: 50 μm for α-synuclein, p-tau, Aβ, and NfL; 5 μm for synaptophysin. Aβ = β-amyloid; BNA = Brainnetome Atlas; DWI = diffusion-weighted imaging; LB = Lewy body; NfL = neurofilament light chain; p-tau = phosphorylated tau.

The workflow of the methods is visualized in Figure 1. Postmortem in situ (brain in cranium) MRI scans were acquired according to a previously described pipeline,^18^ with a maximum postmortem delay (interval between death and autopsy) of 11 hours. In brief, scans were acquired on a 3T scanner (Signa-MR750; General Electric Medical Systems, Chicago, IL) with an 8-channel phased-array head coil. The following pulse sequences were performed for all subjects: (1) sagittal 3D T1-weighted fast spoiled gradient echo (repetition time [TR] = 7 milliseconds, echo time [TE] = 3 milliseconds, flip angle = 15°, 1-mm–thick axial slices, in-plane resolution = 1.0 × 1.0 mm^2^); (2) sagittal 3D fluid attenuation inversion recovery (TR = 8,000 milliseconds, TE = 130 milliseconds, inversion time [TI] = 2,000–2,500 milliseconds, 1.2-mm–thick axial slices, in-plane resolution = 1.11 × 1.11 mm^2^), with TI corrected for postmortem delay; and (3) DWI axial 2D echo-planar imaging with TR/TE = 7,400/92 milliseconds, slice thickness of 2.0 mm, in-plane resolution = 2.0 × 2.0 mm^2^, diffusion gradients applied in 30 non-collinear directions with *b* = 1,000 s/mm^2^, and 5 *b* = 0 s/mm^2^ images. To allow for offline distortion correction of the images, *b*0 images with reversed phase-encoding direction (acquired along anterior-posterior and posterior-anterior directions) were also obtained. To minimize the impact of age-related white matter abnormalities (e.g., vascular change), the 3D-T1 images were lesion-filled^23^ as previously described.^24^

### DWI Preprocessing and Probabilistic Tractography

DWI preprocessing included denoising and removal of Gibbs ringing artifacts, followed by *b*1 field inhomogeneity, eddy current (using oppositely phase-encoded images), and bias field correction, using MRtrix3.^25^ Cortical gray matter was linearly transformed from FreeSurfer into donors' native T1 space and segmented using the Brainnetome Atlas.^26^ Fourteen deep gray matter regions were segmented using FSL's FIRST^27^ and added to the cortical atlas, which together with the cerebellum resulted in a total of 225 regions. The segmented regions were then nonlinearly transformed into donors' native DWI space using Advanced Normalization Tools.^28^ Next, multitissue fiber response functions were produced using the dhollander algorithm and fiber orientation distributions (FODs) through constrained spherical deconvolution with an unsupervised multitissue method and subsequent intensity normalization.^29^ After this, probabilistic tractography was conducted in MRtrix3 using the normalized white matter FODs, by applying iFOD2 probabilistic tracking to generate 10 million streamlines. A hybrid surface/volume segmentation provided anatomical constraints. Finally, streamline weights were calculated using SIFT2,^30^ and a 225 × 225 structural connectivity matrix for each subject was created signifying the weighted number of streamlines connecting each pair of brain regions.

### Graph Theoretical Analysis

The structural connectivity matrix of each patient was thresholded to keep the top 20% strongest connections^31^ and binarized using *in-house* developed Matlab scripts (version R2022B).^32^ Graph theoretical measures were calculated using the Brain Connectivity Toolbox (version 2016_12_09)^4^ and were extracted only for the regions of interest in the right hemisphere from which brain tissue was available.

#### Global Conventional Measures

Global efficiency and mean clustering coefficient across all brain regions (i.e., nodes) were extracted, representing whole-brain integration and segregation, respectively. Global efficiency is defined as the average inverse number of white matter connections (i.e., edges) that need to be traveled to go from one node to the other in the network,^4^ and the clustering coefficient is defined as the fraction of triangles around a node.^4^

#### Regional Conventional Measures

Eigenvector centrality and clustering coefficient were extracted for 8 right-hemisphere cortical regions of interest (ROIs) commonly affected in PD^1,15^ from which brain tissue was available: the superior frontal gyrus, anterior and posterior cingulate gyrus, dorsal anterior insula, middle temporal gyrus, entorhinal cortex, parahippocampal gyrus, and fusiform gyrus (details on the Brainnetome Atlas^26^ labels are provided in eTable 2).

#### Brain Network Resilience

To assess the ability of the brain network to withstand external perturbations, we calculated brain network resilience as the change in global efficiency (ΔGE) after regional node failure of each ROI. This was calculated by subtracting the global efficiency of the intact brain network (GE) from the global efficiency after computationally removing (masking out) a single region from the structural connectivity matrix (GE_lesion_):

ΔGE=GElesion − GE

When ΔGE values are similar to zero, that is, global efficiency after regional node failure is similar to the global efficiency of the intact brain network, high brain network resilience to node failure is indicated. When ΔGE values differ from zero, alterations of global efficiency after regional node failure is indicated, and therefore, a lower brain network resilience to regional node failure.

### Tissue Sampling and Immunohistochemistry

Brain tissue was collected at autopsy. Eight formalin-fixed paraffin-embedded tissue blocks (4%, 4 weeks fixation) of the superior frontal gyrus, anterior and posterior cingulate gyrus dorsal anterior insula (dysgranular), middle temporal gyrus, entorhinal cortex, parahippocampal gyrus, and fusiform gyrus matching the Brainnatome Atlas regions for MRI analysis (eTable 2) were selected. Six-micrometer–thick sections were cut and mounted on superfrost+ glass slides (Thermo Scientific, Waltham, MA). All sections were immunostained for α-synuclein phosphorylated at serine 129 (pSer129 α-synuclein, clone EP1536Y), Aβ (clone 4G8), p-tau (clone AT8), neurofilament light chain (NfL, amino acid sequence 1–376), and synaptophysin (C-terminal). For information on primary antibodies see eTable 3. In brief, brightfield immunostainings were performed for pSer129 α-synuclein, Aβ, p-tau, and NfL. Brightfield immunostainings were visualized with Envision (Dako) and 3.3′-diaminobenzidine (DAB, Dako) with imidazole (50 mg DAB, 350 mg imidazole, and 30 μL of H_2_O_2_ per 100 mL of Tris-HCl 30 mM, pH 7.6), and sections were counterstained with hematoxylin. Fluorescent stainings were performed for synaptophysin, which was visualized with donkey anti-mouse Alexa 488 (eTable 3). Sections were counterstained with 4′,6-diamidino-2-phenylindole (DAPI) (for details, see eMethods).

### Image Analysis

#### Neuropathology Load and Neuroaxonal Degeneration

Using a whole-slide scanner (Vectra Polaris, 20× objective), images of brightfield immunostained sections (pSer129 α-synuclein, Aβ, p-tau, and NfL) were obtained and quantified using QuPath 0.2.3 stardist.^33^ ROIs including cortical layers I–VI were delineated in straight areas to avoid overestimation or underestimation of pathology in sulci and gyri, as described before.^15,16^ The parahippocampal subregions were segmented according to a previously described method,^34^ where the entorhinal cortex, parahippocampal gyrus, and fusiform gyrus were delineated as previously described^15,16^ (segmentation explained in eMethods). As done previously, DAB immunoreactivity was quantified with *in-house* QuPath scripts, using pixel and object classifiers.^15,16^ The outcome measure for pSer129 α-synuclein staining was Lewy body (LB) density (LB count/mm^2^) while outcome measures for Aβ and p-tau stainings were area load (%) (Figure 1C). The outcome measure for NfL staining was area load (%), expressed in the article as immunoreactivity (%).

#### Synaptic Density

Imaging of fluorescent stainings (synaptophysin) was performed using Olympus VS200 (Evident) as previously described.^16^ In brief, a whole-slide overview fluorescence scan was performed using a 10× objective (NA 0.40) on the DAPI channel. Then, 10 ROIs (200 × 160 μm) were drawn, of which 5 in cortical layer III and 5 in cortical layers V-VI within the same cortical minicolumn, corresponding to the superficial and deep pyramidal layers of the cortex, commonly affected in synucleinopathies.^1^ Slides were scanned overnight with a 60× oil objective (NA 1.42) (see eTable 4 for scanning details). Two images were acquired up and down the focus point, with a step size of 0.28 μm in the z-direction. Image preprocessing was performed in Huygens Professional.^35^ Preprocessing consisted of cross-talk correction for subtraction of the autofluorescence signal (such as blood vessels, lipofuscin, and artifacts) from the synaptic channel, and color deconvolution. Subsequently, NIS-elements AR analysis version 5.42.00^36^ was used to quantify synaptophysin^+^ puncta density over neuropil volume. To do so, the neuropil volume was established (excluding nuclei and holes). Subsequently, synaptophysin^+^ puncta were counted with the “bright spots” function, based on average size (0.6 μm) and intensity. The final outcome measure was number of synaptophysin^+^ puncta/μm^3^ per ROI. Finally, outcome measures were averaged first per layer (III and V–VI) and then per region, to obtain 1 synaptophysin^+^ puncta/μm^3^ value per case per region.

### Statistics

Statistical analyses were performed in R-Studio 4.2.1.^37^ Normality was tested, and demographics between groups were compared using Mann-Whitney *U* test for continuous data and Fisher exact test for categorical data. Group differences in pathology load and neuroaxonal and synaptic degeneration were assessed globally with linear mixed models and regionally with nonparametric rank-based estimation tests, both with age and sex as covariates. Group differences in regional MRI measures between PD and control groups were assessed with nonparametric rank-based estimation tests for linear models, with age, sex, and postmortem delay as covariates. To assess which regions contributed to brain network resilience in controls, deviation from zero of regional control ΔGE values was tested. Cohen *d* was calculated to determine effect sizes. False discovery rate (FDR)^38^ correction was applied for multiple testing, after which *p*-values (*p*_FDR_) less than 0.05 were considered significant. Correlations of regional conventional measures (regional eigenvector centrality and regional clustering coefficient) and brain network resilience with regional neuropathology load and neuroaxonal and synaptic degeneration were assessed with rank-based estimation for linear models, with age, sex, and postmortem delay as covariates. FDR correction was applied for multiple marker testing. Correlations of brain network resilience with neuropathologic staging were tested with rank-based estimation for linear models, with age, sex, and postmortem delay as covariates. Correlations of brain network resilience with demographics and clinical data were tested with rank-based estimation for linear models, with postmortem delay as the covariate.

### Data Availability

The data supporting the findings of this study are available from the corresponding author, upon reasonable request.

## Results

### Cohort Description

**Figure 2:**
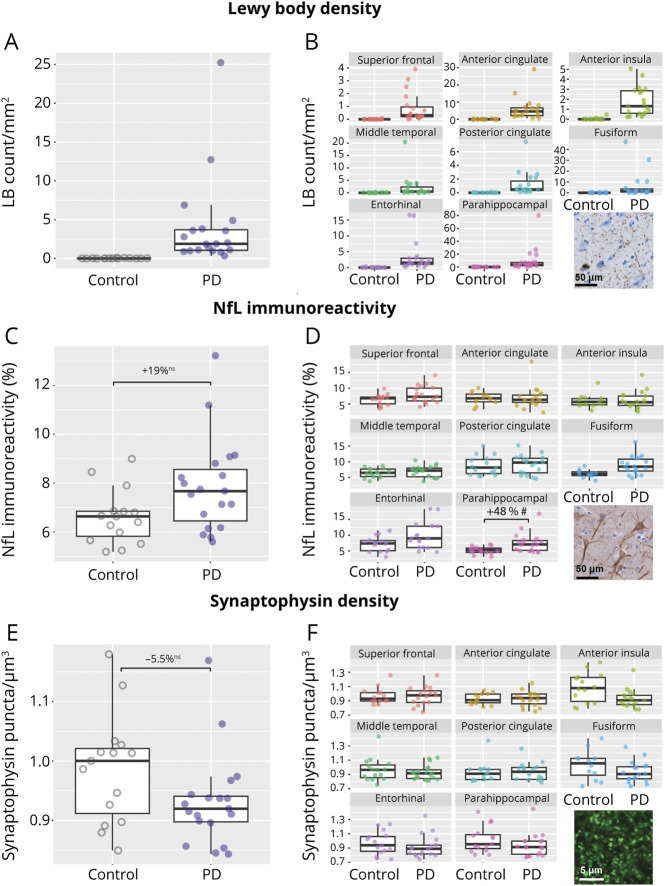
Overall and Regional LB Density, NfL Immunoreactivity, and Synaptophysin Density in Patients With PD and Control Donors A, B show pSer129 α-synuclein–positive LB density (LB count/mm^2^); C, D NfL immunoreactivity (%area); and E, F synaptophysin density (puncta/μm^3^) for control and PD groups. The left column shows the overall measurement across all the cortical regions examined; every data point represents 1 case. The right column shows the regional pathology load for the 8 regions of interest. The immunoreactivity pattern of each marker is shown at the bottom right of each panel. LB density was not tested between patients with PD and controls. For NfL immunoreactivity and synaptophysin density, percentage differences are shown. Ns: nonsignificant, #*p* < 0.05 before FDR correction. FDR = false discovery rate; LB = Lewy body; NfL = neurofilament light chain; PD = Parkinson disease.

Clinical and pathologic data of control and PD donors are summarized in Table 1 (and per donor in eTable 1). Sex (*p* = 0.3) and postmortem delay (*p* = 0.5) did not differ between groups. PD donors were significantly older at death than controls (81 ± 7 years vs 73 ± 9, *p* = 0.009). Regarding copathology, PD donors showed a significantly higher Braak NFT stage compared with controls (*p* = 0.009), but not Thal phase (*p* = 0.400). LB density ranged between 0 and 80 LBs/mm^2^ in PD brain regions (Figure 2, A and B). Cortical Aβ (*p* = 0.288) and p-tau load (*p* = 0.535) did not differ between controls and patients with PD (eFigure 1). NfL immunoreactivity was increased in patients with PD compared with controls in the parahippocampal gyrus (*d* = 0.89, +48%, *p* = 0.040), but significance did not survive multiple testing corrections (*p*_FDR_ = 0.326) (Figure 2, C and D). Average synaptophysin density did not differ between controls and patients with PD in any region (*p* = 0.602) (Figure 2, E and F).

**Table:** Donor Characteristics

	Control	PD	*p* Value
N	15	19 (12 PD, 7 PDD)	—
Sex, male/female (% male)	7/8 (47%)	12/7 (63%)	0.3
Age at symptom onset, y, mean [range]	—	65 [54–84] (2 NA)	—
Disease duration, y, mean [range]	—	16 [8–23] (2 NA)	—
CDR (n total) 0.5/1/2/3 (n)	n = 0 —	n = 11 5/2/2/2 (8 NA)	—
Age at death, y, mean ± SD	73 ± 9	81 ± 7	0.009**
Postmortem delay, h, mean [range]	8.5 [5–11]	8 [3.5–10.5]	0.5
Pathologic characteristics			
Braak α-synuclein stage^1^ 0/1/2/3/4/5/6 (n)	13/1/1/0/0/0/0	0/0/0/1/2/16	—
Thal phase^12^ 0/1/2/3/4/5 (n)	2/8/3/1/1/0	1/6/4/6/2/0	0.4
Braak NFT stage^13^ 0/1/2/3/4/5/6 (n)	3/7/4/1/0/0/0	0/2/10/4/3/0/0	0.009**
LATE-NC stage^50^ 0/1/2/3	15/0/0/0	16/0/3/0	—

Abbreviations: CDR = Clinical Dementia Rating; h = hours; LATE-NC = limbic-predominant age-related TDP-43 encephalopathy neuropathologic change; LB = Lewy body; NA = not available; NFT = neurofibrillary tangle; PD = Parkinson disease; PDD = Parkinson disease dementia; y = years.

Data are mean and range.

*p < 0.05, **p < 0.01, ***p < 0.001.

### Conventional Regional Network Topology Measures Do Not Reflect Neuropathologic Alterations in PD

**Figure 3:**
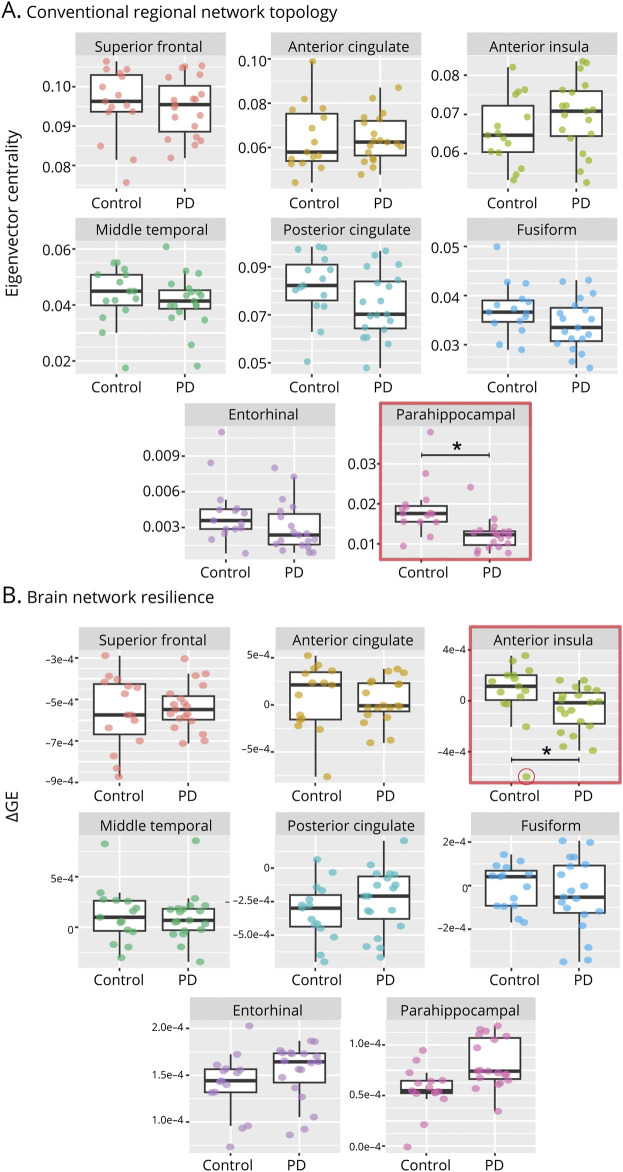
Conventional Regional Network Topology and Brain Network Resilience in Patients With PD and Controls (A) Eigenvector centrality was lower in patients with PD compared with controls in the parahippocampal gyrus. (B) ΔGE was lower in patients with PD compared with controls after regional node failure of the dorsal anterior insula, suggesting that the PD brain network was less resilient to the node failure of the dorsal anterior insula compared with the control brain network. One outlier was present in the control group (encircled in red). ΔGE = change in global efficiency after node failure; GE = global efficiency; PD = Parkinson disease. *p_FDR_ < 0.05.

Global efficiency did not differ between controls and patients with PD (*p* = 0.472) while the global clustering coefficient was significantly higher in patients with PD compared with controls (*d* = 0.59, *p* = 0.018). Regionally, eigenvector centrality was significantly lower in patients with PD compared with controls in the parahippocampal gyrus (*d* = −1.08, *p*_FDR_ = 0.021), but did not differ in other regions (*p*_FDR_ > 0.05, Figure 3A, eTable 5). The clustering coefficient did not differ regionally between patients with PD and controls in any region (*p*_FDR_ > 0.1, eTable 6). Because only eigenvector centrality of the parahippocampal gyrus discerned PD from controls, we explored whether this measure was reflected by regional neuropathologic burden and neuroaxonal and synaptic degeneration. However, no correlations with any neuropathologic outcome measures were found (all *p* > 0.05, eTable 7).

### The PD Brain Network Is Less Resilient to Node Failure of the Dorsal Anterior Insula

ΔGE (a measure of brain network resilience) tended to be lower in patients with PD compared with controls after node failure of the dorsal anterior insula (*d* = −0.65, *p*_FDR_ = 0.064). However, the control group had an outlier (control number 8 in eTable 1), and after this donor was excluded, ΔGE was significantly lower in PD compared with controls (*d* = −1.00, *p*_FDR_ = 0.018) (Figure 3B), indicating a less resilient and less efficient network after node failure of the dorsal anterior insula. ΔGE did not differ between PD and controls after node failure of the other regions (all *p*_FDR_ > 0.1). In controls, node failure of the posterior cingulate and superior frontal cortices showed ΔGE values significantly lower than zero, confirming the importance of these regions for an optimal brain network (for details, see eFigures 2 and 3).

### Lower Brain Network Resilience After Node Failure Does Not Reflect Neuropathologic Alterations Within the Insula or in Connected Regions

In the previous paragraph, we showed that the PD brain network was less resilient and less efficient to the node failure of the dorsal anterior insula compared with the control brain network. Therefore, we set out to explore if any neuropathologic processes within the dorsal anterior insula or in brain areas that are connected to it, such as the anterior cingulate and the entorhinal cortex,^39^ were driving the decrease in PD brain network resilience.

#### Pathology Within the Insula

In PD, LB density, Aβ load, p-tau load, NfL immunoreactivity, and synaptophysin density in the dorsal anterior insula did not correlate with ΔGE after node failure of the dorsal anterior insula (all *p*_FDR_ > 0.1). Across the whole cohort, similar results were found (all *p*_FDR_ > 0.1).

#### Pathology Within the Anterior Cingulate Cortex

In PD, LB density, Aβ load, p-tau load, NfL immunoreactivity, and synaptophysin density within the anterior cingulate cortex did not correlate with ΔGE after node failure of the dorsal anterior insula (all *p*_FDR_ > 0.1). Across the whole cohort, similar results were found (all *p*_FDR_ > 0.1).

#### Pathology Within the Entorhinal Cortex

In PD, LB density, Aβ load, p-tau load, NfL immunoreactivity, and synaptophysin density within the entorhinal cortex did not correlate with ΔGE after node failure of the dorsal anterior insula (all *p*_FDR_ > 0.1). Across the whole cohort, Aβ load within the entorhinal cortex tended to correlate with ΔGE after node failure of the dorsal anterior insula (*r*_s_ = −0.38, *p*_FDR_ = 0.054). No correlations were found for the other markers (all *p*_FDR_ > 0.1).

### Lower Brain Network Resilience After Node Failure Reflects Global Braak α-Synuclein Staging

In the previous paragraph, we showed that the decreased resilience of the PD brain network to node failure of the dorsal anterior insula was not driven by neuropathologic processes occurring within the dorsal anterior insula or in regions connected to it. Therefore, we set out to explore whether specific demographics and clinical data or global neuropathologic stages were driving the decrease in brain network resilience in PD.

#### Demographics and Clinical Data

In the PD group, sex (*p*_FDR_ = 1.000), disease duration (*p*_FDR_ = 0.672), CDR score (*p*_FDR_ = 1.000), and age at death (*p*_FDR_ = 1.000) did not correlate with ΔGE after node failure of the dorsal anterior insula. Across the whole cohort, ΔGE after node failure of the dorsal anterior insula did not correlate with sex (*p*_FDR_ = 1.000) and age at death (*p*_FDR_ = 1.000).

#### Neuropathologic Staging

ΔGE after node failure of the dorsal anterior insula negatively correlated with Braak α-synuclein stages across the combined cohort (*r*_s_ = −0.40, *p*_FDR_ = 0.036; eFigure 4), but did not correlate with Braak NFT stage (*p*_FDR_ = 0.344) or Thal phase (*p*_FDR_ = 1.000).

Taken together, our results show that decreasing resilience of the PD brain network to node failure of the dorsal anterior insula correlated with higher global Braak α-synuclein staging.

## Discussion

In this study, we investigated whether neuropathologic processes contribute to brain regional topologic properties of integration and segregation and brain network resilience in PD, by using a combined within-subject postmortem MRI and histopathology approach. Using conventional regional network topology measures, we found a lower eigenvector centrality in the parahippocampal gyrus, but this was not related to regional pathologic accumulations. Using the more advanced measure of brain network resilience, we found that the brain network of PD donors was less resilient to node failure of the dorsal anterior insula compared with the control brain network. Neuropathologic processes within the dorsal anterior insula itself or within brain areas that are connected to it did not directly drive this change, but seemed related to a global increase in α-synuclein.

With conventional regional network topology measures, we found lower regional eigenvector centrality in the parahippocampal gyrus in patients with PD compared with controls and no difference in clustering coefficient. The parahippocampal gyrus was reported to be part of a subnetwork with lower connectivity in de novo PD,^6^ which also involved several striatal and limbic regions, suggesting that the parahippocampal gyrus is affected already in early disease stages. However, the lower eigenvector centrality of the parahippocampal gyrus did not correlate with regional pathologic load and neuroaxonal and synaptic degeneration. While regional topologic changes in the brain network are usually hypothesized to be driven by regional pathologic processes, such as neuronal and synaptic loss, our results indicate that this is likely not the case.

We found that the brain network of PD donors was less resilient to node failure of the dorsal anterior insula compared with the control brain network. The human brain is highly resistant to targeted and random attacks, by virtue of how the structural brain network is organized.^8,40^ However, this ability has yet to be investigated in PD. Only 1 study investigated brain resilience in functional networks of patients with PD using resting-state functional MRI in combination with targeted attacks^41^ and found that the resilience of the frontoparietal network was associated with the absence of cognitive decline in PD. In our study, we took a different approach to assess brain resilience: first, by using structural rather than functional network topology and second, by using the change in global efficiency after masking out key regions affected in PD, rather than targeted or random attacks (i.e., targeted removal of hub regions, or random removal of regions), similar to studies in the context of stroke.^42^ By using this approach, we found that the PD brain network is not as resilient as the control brain network to the failure of the dorsal anterior insula, suggesting that this region might acquire a more central role in the structural connectome of patients with PD. The dorsal anterior insula has been shown to have an increased eigenvector centrality in patients with PD compared with non-demented controls.^43^ Moreover, the salience network, which includes the insula as the hub region, was shown to have structural and functional alterations in PD, which associated with depression and cognitive impairment.^44,45^

With our postmortem MRI and pathology approach, we have shown that decreased resilience of the PD brain network to node failure of the dorsal anterior insula was not driven by neuropathologic processes such as pathology load and neuroaxonal or synaptic degeneration within the dorsal anterior insula itself or within brain areas connected to it. Our results are in line with previous studies that have consistently shown that the effect of a lesion is not only driven by its topologic location but importantly also by the global organizational structure of the brain network.^46^ Translating this back to neurodegenerative diseases, our results suggest that the fact that the dorsal anterior insula becomes more central within the PD network is probably not because of the regional pathologic processes within the dorsal anterior insula itself but more likely because of the global effect of α-synuclein pathology on the reorganization of the whole connectome. Supporting this theory, we found that decreased resilience of the PD brain network to node failure of the dorsal anterior insula was associated with higher Braak α-synuclein staging. In fact, at high Braak stages, not only a vast number of regions are affected by LB pathology but also regions affected at earlier stages show higher pathologic burden.^1^

The strength of this study lies in the combined within-subject postmortem MRI and histopathologic data spanning from common neuropathologic accumulations to neuroaxonal and synaptic loss, which are commonly hypothesized substrates of MRI regional topologic changes. However, there are a few limitations that are worth mentioning. First, although we included a total of 34 brain donors with both postmortem MRI and histopathology, the sample size is limited and might suffer from selection bias because of the donation programs, inevitably introducing heterogeneous disease duration and advanced disease stage. Clinical information of the donors was also limited (including no reliably collected years of education), and CDR scores were available only for a part of the PD donors. In addition, one could speculate that brain network resilience may vary with disease stage, with a more active response to PD pathology at early stages and a more passive response at later stages. To explore this further, an ideal study design would include PD donors at different stages of disease. The correlation between brain network resilience and Braak α-synuclein staging should be interpreted with caution because most of the cases were either controls (i.e., Braak stage 0) or advanced PD (i.e., Braak stage 6), and only 5 cases showed intermediate Braak α-synuclein stages (although their data points fall close to the regression line). Moreover, although postmortem DWI was acquired in situ, several factors (such as lower body temperature, tissue decomposition, swelling, hypoxia, and partial volume effects) might have influenced diffusivity properties.^47^ To minimize these effects, we took into account postmortem delay as a covariate in the analyses, but this should be kept in mind when comparing our results with those of in vivo studies. Finally, even if one of our aims was to investigate brain network resilience in PD, this measure could also be interpreted as brain network robustness because the 2 terms share a robust conceptual overlap,^48^ and as connectomal diaschisis because our measure also describes the remote effects of a lesion on the structural brain connectome.^49^

The brain network of PD donors was less resilient to node failure of the dorsal anterior insula compared with that of controls; however, this was not driven by any neuropathologic processes within the dorsal anterior insula itself or in connected brain areas but more by an overall global increase in α-synuclein accumulation. Therefore, our results highlight that regional network disturbances are more complex to interpret than previously believed and that PD neuropathology probably has a widespread effect on brain network reorganization rather than regional effects on topologic measures.

## Appendix Authors

**Table:**

Name	Location	Contribution
Irene Frigerio, MSc	Department of Anatomy and Neurosciences, Amsterdam UMC location Vrije Universiteit Amsterdam, the Netherlands	Drafting/revision of the manuscript for content, including medical writing for content; major role in the acquisition of data; study concept or design; analysis or interpretation of data
Tommy A.A. Broeders, MSc	Department of Anatomy and Neurosciences, Amsterdam UMC location Vrije Universiteit Amsterdam, the Netherlands	Major role in the acquisition of data
Chen-Pei Lin, MSc	Department of Anatomy and Neurosciences, Amsterdam UMC location Vrije Universiteit Amsterdam, the Netherlands	Major role in the acquisition of data
Maud M.A. Bouwman, MSc	Department of Anatomy and Neurosciences, Amsterdam UMC location Vrije Universiteit Amsterdam, the Netherlands	Major role in the acquisition of data
Ismail Koubiyr, PhD	Department of Anatomy and Neurosciences, Amsterdam UMC location Vrije Universiteit Amsterdam, the Netherlands	Major role in the acquisition of data
Frederik Barkhof, MD, PhD, FRCR	Department of Radiology and Nuclear Medicine, Amsterdam UMC location Vrije Universiteit Amsterdam, the Netherlands; Institutes of Neurology and Healthcare Engineering, University College London, United Kingdom	Analysis or interpretation of data
Henk W. Berendse, MD, PhD	Department of Neurology, Amsterdam UMC location Vrije Universiteit Amsterdam, the Netherlands	Analysis or interpretation of data
Wilma D.J. Van De Berg, PhD	Department of Anatomy and Neurosciences, Amsterdam UMC location Vrije Universiteit Amsterdam, the Netherlands	Study concept or design; analysis or interpretation of data
Linda Douw, PhD	Department of Anatomy and Neurosciences, Amsterdam UMC location Vrije Universiteit Amsterdam, the Netherlands	Study concept or design; analysis or interpretation of data
Laura E. Jonkman, PhD	Department of Anatomy and Neurosciences, Amsterdam UMC location Vrije Universiteit Amsterdam, the Netherlands	Study concept or design; analysis or interpretation of data

## Glossary

ΔGE: change in global efficiency after node failure
Aβ: β-amyloid
AD: Alzheimer disease
CDR: Clinical Dementia Rating
DAB: 3.3′-diaminobenzidine
DAPI: 4′,6-diamidino-2-phenylindole
DWI: diffusion-weighted imaging
FDR: false discovery rate
FOD: fiber orientation distribution
GE: global efficiency
LB: Lewy body
NABCA: Normal Aging Brain Collection Amsterdam
NBB: Netherlands Brain Bank
NfL: neurofilament light chain
NFT: neurofibrillary tangles
PD: Parkinson disease
p-tau: phosphorylated tau
ROI: region of interest
TE: echo time
TI: inversion time
TR: repetition time
WM: white matter
